# Electrochemically Induced Oxide‐to‐Hydroxide Transformation Enables Fast Proton Transport for Enhanced Hydrogen Evolution

**DOI:** 10.1002/advs.75242

**Published:** 2026-04-09

**Authors:** Jiaying Mo, Lingling Zhai, Alex W. Robertson, Chiu C. Tang, Sarah J. Day, Simson Wu, Lu Chen, Tsz Woon Benedict Lo, Molly Meng‐Jung Li, Shu Ping Lau, Xin‐Ping Wu, Yiyang Li, Shik Chi Edman Tsang

**Affiliations:** ^1^ Wolfson Catalysis Centre Department of Chemistry University of Oxford Oxford UK; ^2^ Department of Applied Physics The Hong Kong Polytechnic University Hung Hom Hong Kong P. R. China; ^3^ Department of Materials University of Oxford Oxford UK; ^4^ Department of Physics University of Warwick Coventry UK; ^5^ Diamond Light Source Rutherford Appleton Laboratory Didcot UK; ^6^ State Key Laboratory of Green Chemical Engineering and Industrial Catalysis Centre for Computational Chemistry and Research Institute of Industrial Catalysis School of Chemistry and Molecular Engineering East China University of Science and Technology Shanghai P. R. China; ^7^ State Key Laboratory of Chemical Biology and Drug Discovery Department of Applied Biology and Chemical Technology The Hong Kong Polytechnic University Hung Hom Hong Kong P. R. China

**Keywords:** electrocatalysis, hydrogen evolution, magnesium oxide, single atom catalysts, topotactic phase change

## Abstract

Developing earth‐abundant electrocatalysts that rival the commercial platinum/carbon catalyst for the hydrogen evolution reaction (HER) remains a central challenge in renewable‐energy conversion. Here, we reveal an electrochemically induced, in situ phase transformation in a Ru‐MgO catalyst that leads to true active material during operation. Under acidic HER conditions, nominal 20 wt.% Ru nanoparticles supported on polar MgO(111) nanocrystals undergo a topotactic hydrolysis to Ru‐Mg(OH)_2_(001), generating an ordered hydroxide layer that serves as a highly conductive proton‐hopping network. After activation, the catalyst delivers performance comparable to commercial Pt/C under identical conditions, matching the current density of −1.1 V and surpassing it by approximately 10% at −2.3 V. *Operando* synchrotron X‐ray diffraction combined with ex situ characterization techniques directly captures this transformation, while density‐functional theory calculations reveal that water‐assisted Grotthuss proton transfer across the hydroxide requires only a 0.10 eV energy barrier. These findings establish electrochemically driven oxide‐to‐hydroxide conversion as a new design principle for creating low‐Pt or Pt‐free HER electrocatalysts with intrinsically fast proton transport.

## Graphical abstract

An electrochemically induced transformation of polar MgO(111) into Mg(OH)_2_(001) creates a proton‐conductive hydroxide layer during hydrogen evolution. Operando synchrotron XRD and DFT reveal a low‐barrier Grotthuss proton‐hopping mechanism, establishing a feasible strategy for designing oxide‐hydroxide electrocatalysts with enhanced proton transport.

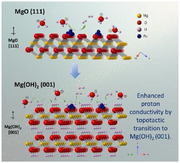

## 1 Introduction

Heterogeneous electrocatalysts for the hydrogen‐evolution reaction (HER) typically rely on finely divided noble metals dispersed on oxide supports to maximize activity while minimizing precious‐metal usage. Beyond serving as inert carriers, oxide supports can profoundly influence the structure and electronic properties of the active metal through strong metal‐support interactions (SMSI), thereby tuning catalytic performance [1, 2].

Among various metal oxide supports, non‐redox magnesium oxide (MgO) is particularly attractive owing to its chemical stability and tunable surface chemistry [3, 4]. Morphology‐dependent effects of MgO have been documented across diverse catalytic reactions [5, 6, 7], yet its potential in electrochemical catalysis remains largely untapped. Of special interest is the high‐energy polar MgO(111) facet, which consists of alternating O^2−^ and Mg^2+^ layers, creating distinct O‐ and Mg‐terminated surfaces. By contrast, the MgO(110) and MgO(100) surfaces present mixed, non‐polar terminations with only isolated active sites [8]. The unusual stability of the polar (111) surface arises mainly from interactions between filled O^2−^ 2p and vacant Mg^2+^ orbitals moderated by the Madelung potential. Preferential exposure of {111} planes lowers the energy of the Mg^2+^ orbitals while raising that of O^2−^, reducing electron‐binding energies and enabling new electronic states [9]. To compensate for the high surface energy, such polar facets typically relax via defect formation, surface reconstruction, or adsorption of charge‐balancing species. For non‐redox MgO(111), adsorption of counter‐ions is favored because defect formation is energetically costly [9, 10].

Recent work by Wu et al. demonstrated that single Ru^2+^ atoms dispersed on MgO(111) catalyze reversible hydrogen conversion through Ru^2+^‐O^2−^ frustrated Lewis pairs [3, 4]. This discovery suggests that the MgO(111) surface may play an active role in proton management, making it an intriguing yet scarcely explored support for aqueous HER. Platinum remains the benchmark HER catalyst, typically used as 20 wt.% Pt/C, because of its optimal hydrogen‐binding energy and high intrinsic activity [11, 12]. However, its scarcity and cost hinder large‐scale deployment. Ruthenium offers a promising alternative: it shares similar hydrogen‐binding strength (≈65 kcal mol^−1^), exhibits a lower water‐dissociation barrier, and costs roughly 4% as much as Pt [13, 14]. Considerable efforts have therefore focused on reducing noble‐metal content and exploiting SMSI to enhance HER performance. Although metal oxides provide chemical and electrochemical stability, their poor electronic conductivity typically limits activity [15, 16, 17]. Approaches to overcome this drawback include forming conductive composites or tailoring exposed crystal facets to increase surface conductivity and proton mobility [18, 19, 20, 21, 22]. The polar MgO(111) surface is especially intriguing in this context. Its high proton affinity, coupled with the close spacing of surface oxygen atoms, can stabilize adsorbed hydrogen and promote proton migration through a hydrogen‐bonded network via a Grotthuss‐type mechanism [23]. Yet, despite these promising attributes, the electrochemical behavior of MgO(111) under HER conditions and its potential to undergo structural evolution that benefits proton transport remain virtually unexplored.

Herein, we address this gap by investigating ruthenium nanoparticles supported on MgO with different preferentially exposed facets, including (111), (110), and (100), as cathode catalysts for acidic HER. We show that Ru/MgO(111) undergoes an electrochemically driven topotactic transformation to Ru‐Mg(OH)_2_(001), creating efficient proton‐hopping channels. After activation, this catalyst delivers a hydrogen‐evolution current density exceeding that of commercial Pt/C by approximately10% at ‐2.3 V. Using in situ synchrotron X‐ray diffraction (XRD) and X‐ray photoelectron spectroscopy (XPS), together with ex situ TEM and SAED, we reveal how the Ru‐MgO(111) interface evolves under reaction conditions and demonstrate that the resulting Ru‐Mg(OH)_2_(001) phase functions as a proton‐conducting support. Our findings highlight how in situ probing of electrochemical phase transitions can uncover design principles for durable, high‐performance HER catalysts and inspire the development of Pt‐efficient or Pt‐free electrochemical technologies.

## 2 Results and Discussion

**FIGURE 1:**
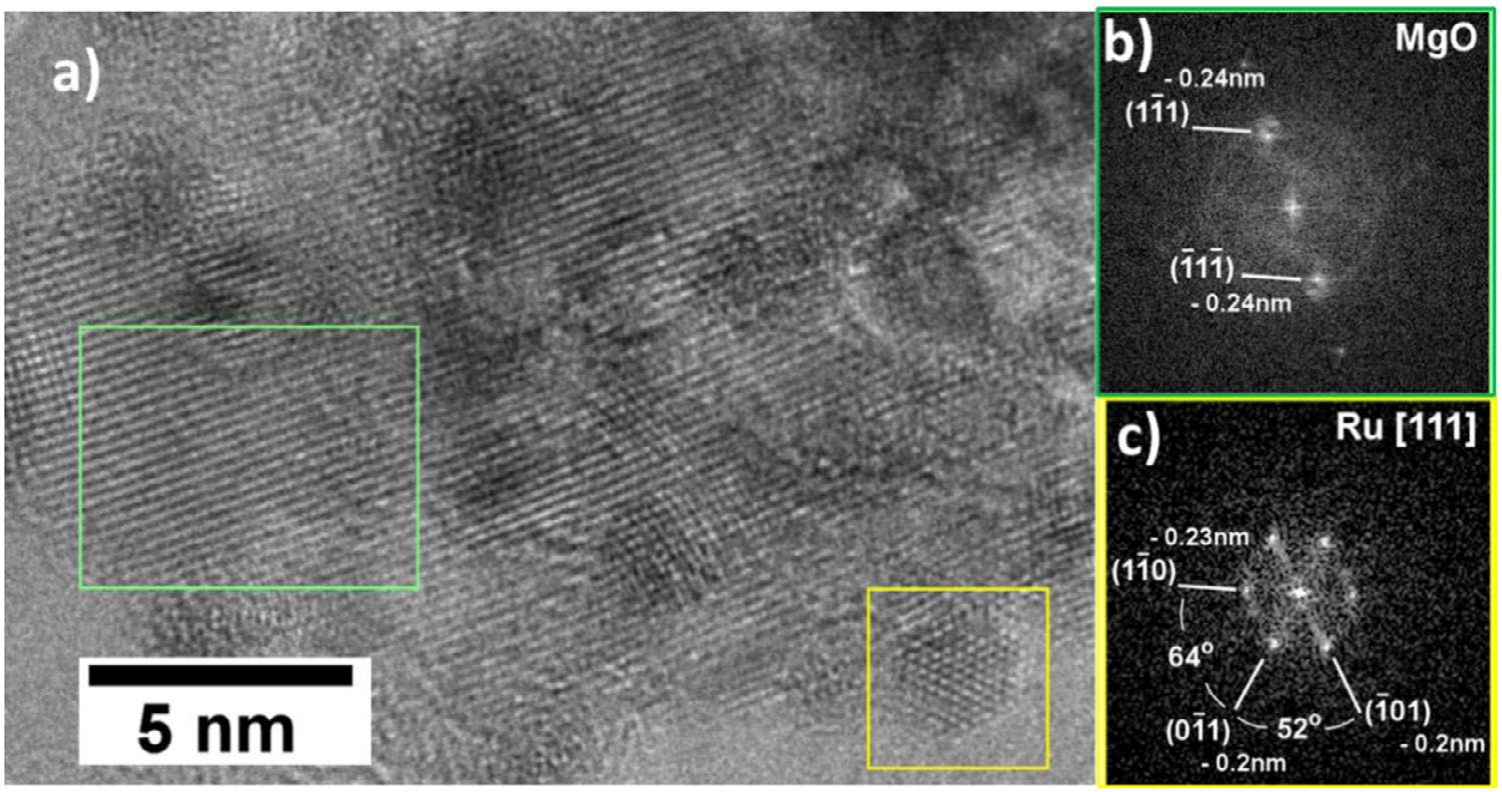
(a) HRTEM of the nominal 20 wt.% Ru‐MgO (111) sample with the corresponding FFT patterns of faceted MgO (111) region in the green box (b) and Ru NP region in the yellow box (c).

MgO supports with preferentially exposed (111), (110), and (100) facets were synthesized for HER studies (Note S1). X‐ray diffraction (XRD) patterns of the three samples (Figure S1) confirmed the rock‐salt periclase structure of MgO [24], with the (200) reflection showing the highest intensity in all cases. This dominance arises because XRD primarily probes bulk crystallinity, and the nanoscale nature of the powders leads to randomly oriented grains where the thermodynamically stable (200) facet diffracts most strongly, regardless of the dominant exposed surface. In contrast, transmission electron microscopy (TEM) provided direct insight into surface morphology and lattice spacing (Figure S2). MgO(111) exhibited hexagonal nanosheets with a spacing of 0.243 ± 0.010 nm, consistent with literature values [25, 26]. MgO(110) formed stacked leaf‐like structures with 0.148 ± 0.008 nm fringes corresponding to exposed (220) planes [26, 27], while MgO(100) displayed amorphous‐like structures dominated by the stable (100) facet, giving a spacing of 0.213 ± 0.010 nm (Figure S2f) [5]. Surface area analysis by BET (Table S1) showed MgO(110) possessed the largest area (214.1 m^2^ g^−^^1^), followed by MgO(111) (170.9 m^2^ g^−^^1^), with MgO(100) significantly lower at 13.4 m^2^ g^−^^1^. This trend, (100) < (111) < (110) correlates with crystallite size inferred from XRD. Given its markedly lower surface area, MgO(100) was deemed unsuitable for fair electrochemical comparison; therefore, Ru nanoparticles were loaded only onto MgO(111) and MgO(110) supports for subsequent investigations. Figure 1 presents HR‐TEM images of the as‐prepared nominal 20 wt.% Ru NPs supported on MgO(111) nanosheets. The HR‐TEM image in Figure 1a shows that the dominant exposed facets remain (111) without noticeable surface reconstruction, as further confirmed by the fast Fourier transform (FFT) pattern in Figure 1b. The measured lattice spacing of 0.24 nm matches well with that of pristine MgO(111) (Figure S2d), indicating that the preferential (111) surface is preserved during the sono‐chemical Ru loading process. In addition, the FFT pattern in Figure 1c displays the characteristic reflections of a crystalline Ru nanoparticle. A comprehensive determination of the exposed facets has been reported in our previous work, employing probe‐assisted nuclear magnetic resonance spectroscopy to identify different surface facets [3, 4].

**FIGURE 2:**
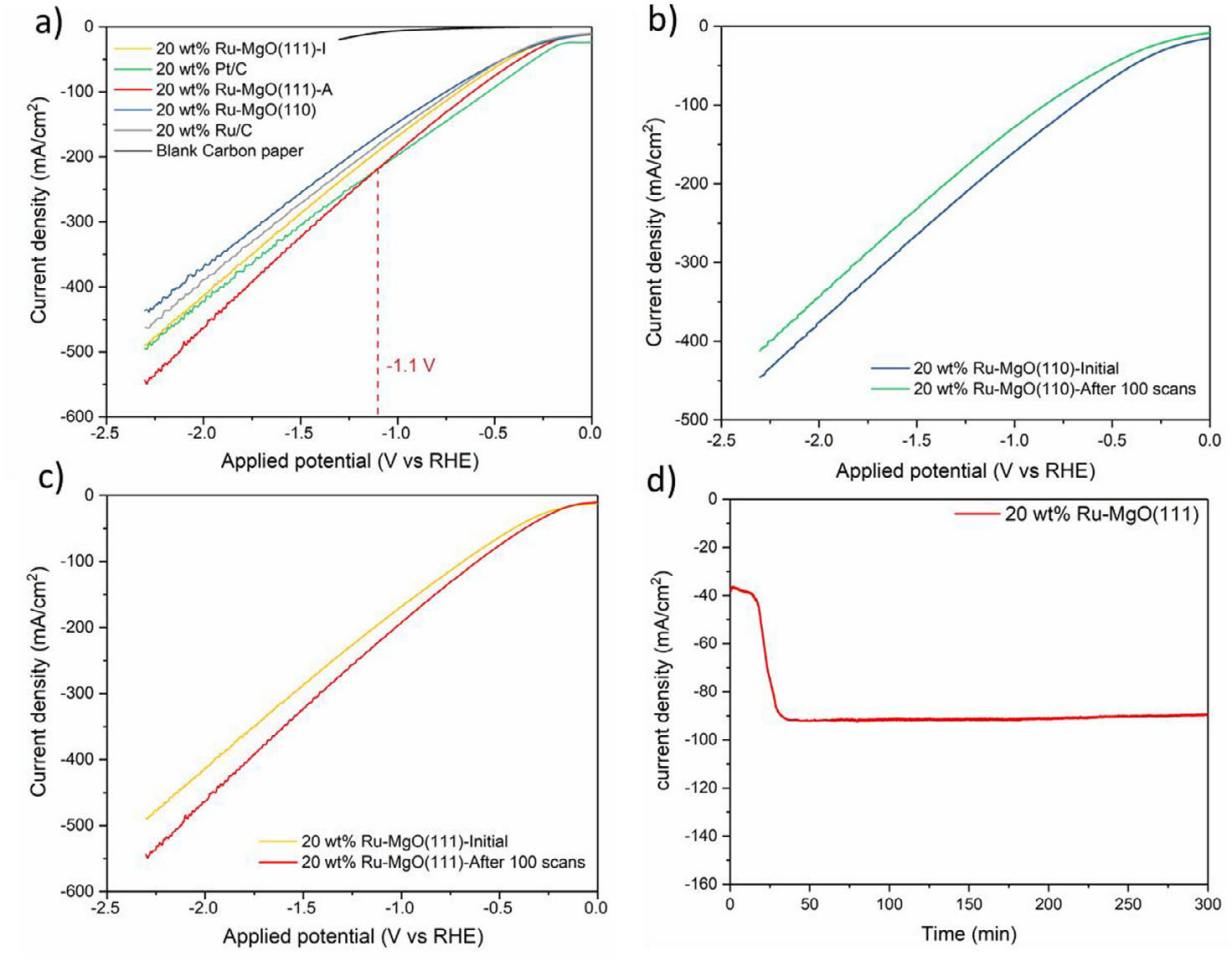
(a) LSV polarization curves of the as‐prepared Ru‐MgO (110), Pt/C, Ru/C, Ru‐MgO (111), and blank carbon paper in 0.5 M H_2_SO_4_. I (initial) and A (after) are corresponding to the Ru‐MgO (111) catalysts before and after 100 scans respectively; (b) LSV polarization curves before and after 100 scans on Ru‐MgO (110); (c) LSV polarization curves before and after 100 scans on Ru‐MgO (111); (d) Chronoamperometric curve of the Ru‐MgO (111) at −1.8 V for 300 min in 0.05 M H_2_SO_4_ electrolyte.

The hydrogen evolution activity of nominal 20 wt.% Ru supported on MgO(111) and MgO(110), as well as Ru/C and commercial Pt/C (nominal 20 wt.%), was assessed by linear sweep voltammetry (LSV) in 0.5 M H_2_SO_4_ using a conventional three‐electrode configuration (Figure 2a). Polarization curves were collected at a scan rate of 5 mV s^−1^ following ten preliminary CV cycles at 50 mV s^−1^. Here, the catalysts are denoted according to their nominal synthesis loading (20 wt.%), whereas the actual metal contents determined by ICP‐MS are 14.2 wt.% for Ru‐MgO(111), 15.7 wt.% for Ru‐MgO(110), 20.2 wt.% for commercial Pt/C, and 21.4 wt.% for Ru/C (Table S1). In its initial state, Ru‐MgO(111) exhibited lower current densities than Pt/C; however, after 100 potential scans (denoted as Ru‐MgO(111)‐A), the catalyst underwent a pronounced activation, achieving activity comparable to Pt/C up to −1.1 V and surpassing it at more negative potentials. At −2.3 V, Ru‐MgO(111)‐A reached a current density of 548 mA cm^−^^2^, approximately 10% higher than Pt/C (495 mA cm^−2^), despite the latter containing a higher noble‐metal loading and a higher support surface area (Tables S1and S2). To account for the deviation between nominal and actual metal loading, the HER activity was further normalized to the ICP‐determined noble‐metal mass (mA mg_metal_^−1^). After normalization, activated Ru‐MgO(111) retains higher mass activity than commercial Pt/C (Table S3), confirming that its superior HER performance remains valid when the actual noble‐metal loading is considered.

By contrast, Ru‐MgO(110) undergoes progressive performance decay upon repeated cycling (Figure 2b) and during chronoamperometric testing (Figure S3). In comparison, Ru‐MgO(111) displayed a continuous increase in current density during operation, rising from −36.8 to −91.8 mA cm^−2^ within the first 35 min (i.e., an enhancement of 149.5%), before stabilizing at a high steady‐state level (Figure 2c,d). These observations suggest that Ru‐MgO(111) undergoes an electrochemical activation process under HER conditions, transforming into a highly active and stable catalytic phase, whereas Ru‐MgO(110) gradually deactivates.

**FIGURE 3:**
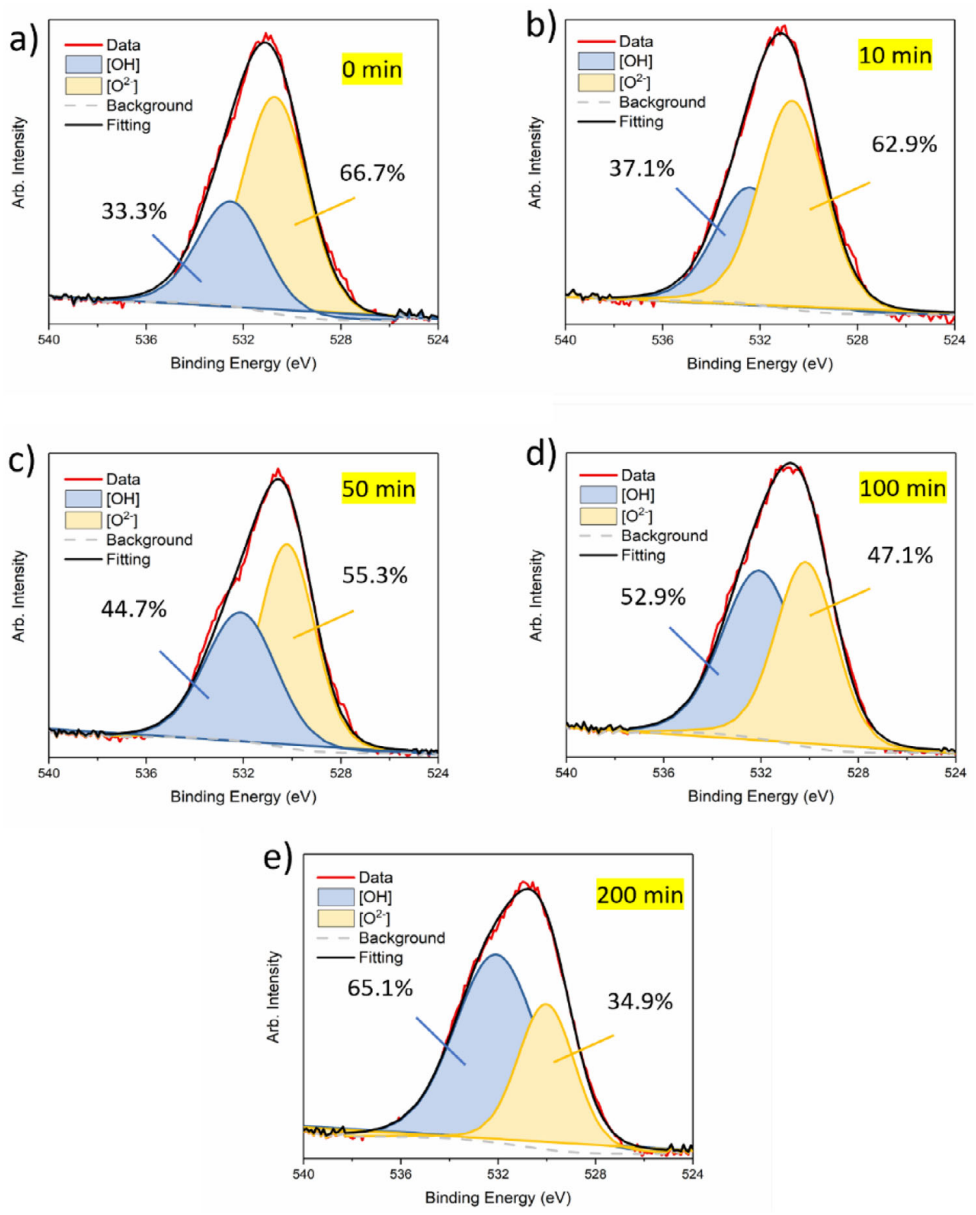
XPS profile of O 1s region at different time (a) 0, (b) 10, (c) 50, (d) 100, and (e) 200 min of the reaction. The blue shaded area is the [OH] component and the yellow shaded area is the [O^2−^] component.

To elucidate the unusual activation behavior of Ru‐MgO(111) during HER, extensive characterization was performed. XPS was employed to probe the surface chemical environment, with all samples pre‐etched by Ar^+^ ions for 60 s to remove surface contamination. Because the C 1s signal, which is conventionally used as the calibration, overlaps with Ru 3d, all spectra were thus calibrated against the Mg 2s peak at 88.1 eV [28]. As shown in Figure 3, the O 1s region can be deconvoluted into OH species at 531–532.5 eV and O^2−^ species at 529–530 eV (Table S4) [28]. Notably, a substantial fraction of OH (∼33%) was already present at the initial stage (0 min), likely due to proton stabilization on the oxygen‐terminated polar facets generated during synthesis. With increasing electrochemical reduction time, the OH contribution progressively increased while the O^2−^ signal decreased proportionally. After approximately 3 h of reaction, the OH fraction nearly doubled (from 33.3% to 65.1%), indicating extensive proton incorporation at the MgO surface. In contrast, a control experiment performed without applied potential showed no change in the OH content. These results suggest that the unusually high OH fraction arises from electrochemical proton‐driven hydrolysis of MgO(111), extending beyond the topmost surface layer.

To monitor structural evolution under reaction conditions, *operando* electrochemical synchrotron XRD (SXRD) measurements were conducted in a specially designed three‐electrode cell (Figure S4). Data were collected during chronoamperometry at −1.8 V (Figure 2d), with the electrolyte diluted to 0.05 M H_2_SO_4_ to minimize background scattering.

**FIGURE 4:**
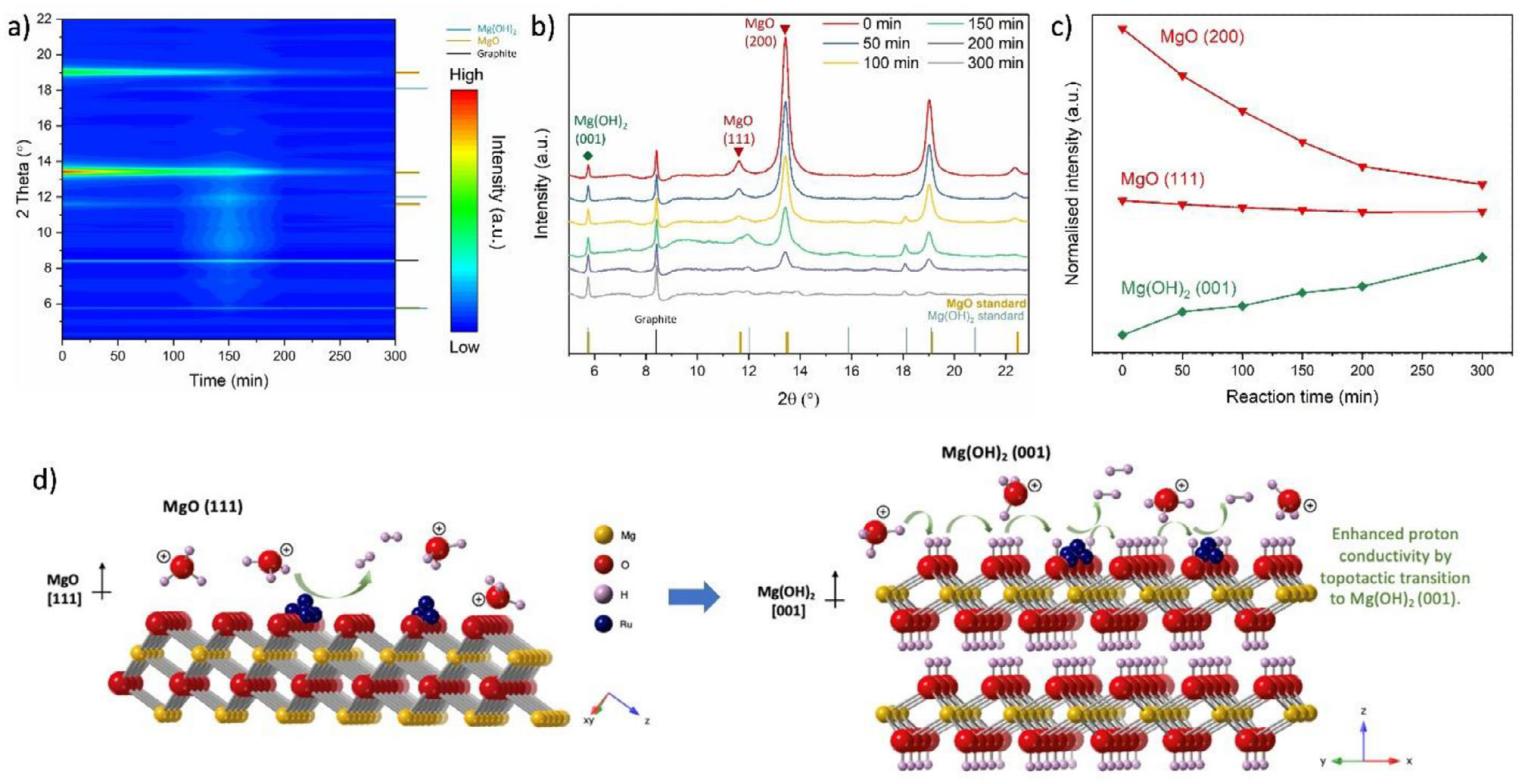
(a) In situ background‐subtracted contour plot and (b) SXRD patterns of the Ru‐MgO (111) sample under −1.8 V with the structural change to Mg(OH)_2_. The reference diffraction patterns of MgO and Mg(OH)_2_ are shown at the bottom for comparison. (c) The corresponding normalized intensity variations of MgO (200), Mg0 (111) and Mg(OH)_2_ (001) as the reaction progresses. (d) Schematic representations of the topotactic transitions from MgO to Mg(OH)_2_ via electrochemical hydrolysis on MgO (111) supports. The hydronium ions are reduced on the Ru clusters which are supported on the MgO or Mg(OH)_2_.

Comparison of the *operando* patterns with reference diffractograms of MgO and Mg(OH)_2_ (Figure 4a,b; Figure S5) revealed a clear in situ transformation from cubic MgO to hexagonal Mg(OH)_2_ during HER. As the current density increased from −36.8 to −91.8 mA cm^−2^ within the first 35 min (Figure 2d), the intensities of MgO reflections at 11.7° (111), 13.5° (200), 19.0° (220), and 22.5° (311) gradually diminished. Concurrently, new peaks emerged at 5.8°, 12.0°, 15.9°, and 18.1°, corresponding to the (001), (101), (102), and (110) reflections of Mg(OH)_2_ (Figure 4b; Figure S6). This progression indicates that Mg(OH)_2_ formation occurs at the expense of MgO, with the graphite peak at 8.3° (from the carbon paper) remaining unchanged. Notably, overlap exists between MgO (220) and Mg(OH)_2_ (110) at ∼19.0°, and MgO (101) and Mg(OH)_2_ (001) at ∼5.8°, but intensity changes confirm the growth of Mg(OH)_2_ (001) alongside the decay of MgO (220).

Quantitative tracking of peak intensities (Figure 4c) further supports this structural transition: MgO(111) and MgO(200) reflections decrease gradually while Mg(OH)_2_ (001) grows throughout the 300‐min reaction. The crystallographic relationship (Figure 4d) indicates a topotactic conversion in which MgO(111) is parallel to Mg(OH)_2_(001), and MgO [1$\bar{1}$0] is parallel to Mg(OH)_2_[110] [25, 29, 30]. This suggests that the MgO unit cell expands along [111] under electrochemical hydrolysis, driving a transition into hexagonal Mg(OH)_2_. Initially, Ru clusters on MgO(111) provide active sites for hydronium (H_3_O^+^) reduction. As the reaction proceeds, protons penetrate the (111) planes, forming Mg─OH bonds between Mg layers and generating <001>‐oriented Mg(OH)_2_ nanosheets. The resulting Mg(OH)_2_(001) surfaces, rich in hydroxyl groups, facilitate proton transport via hydrogen‐bond–mediated hopping, thereby enhancing proton conductivity and accelerating reduction at Ru sites. This topotactic transformation directly correlates with the observed increase in current density during activation.

Furthermore, a control experiment confirmed that the transformation is electrochemically driven: Ru‐MgO(111) samples immersed in electrolyte for 12 h without applied potential showed no structural changes or new diffraction peaks (Figure S7). In contrast, Ru‐MgO(110) exhibited a markedly different response under identical operando conditions. Here, current density decreased steadily with time (Figure S3), and SXRD patterns (Figures S8 and S9) retained sharp MgO reflections with only minor intensity loss in (200) and (220), consistent with crystal collapse under potential stress. No Mg(OH)_2_ peaks were detected, underscoring the unique propensity of MgO(111) to undergo electrochemically induced topotactic conversion into Mg(OH)_2_(001).

**FIGURE 5:**
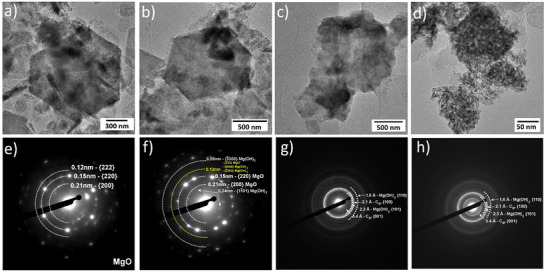
TEM images of the Ru‐MgO (111) after (a) 0, (b) 10, (c) 50, and (d) 200 min of reaction, and (e–h) the corresponding SAED patterns.

To further verify the structural and compositional evolution of Ru‐MgO(111) during HER, ex situ TEM was then performed on samples collected at 0, 10, 50, and 200 min under −1.8 V in 0.5 M H_2_SO_4_. The as‐prepared sample displayed well‐defined hexagonal nanosheets with smooth, flat surfaces (Figure 5a), and the corresponding SAED pattern (Figure 5e) showed sharp diffraction spots, confirming high crystallinity. After 10 min of reaction, the nanosheet morphology was largely preserved (Figure 5b), while the SAED pattern (Figure 5f) revealed MgO as the dominant phase with emerging Mg(OH)_2_ reflections, consistent with the early stages of the MgO to Mg(OH)_2_ transition observed by operando SXRD (Figure 4).

At 50 min, the nanosheets developed coarse and rough surfaces with noticeable distortion of the hexagonal framework (Figure 5c). The associated SAED pattern (Figure 5g) exhibited diffuse rings, indicating the growth of multiple small Mg(OH)_2_ domains and a loss of long‐range crystallinity [31]. The presence of faint graphite rings was attributed to contamination from the graphite counter electrode. After 200 min, the nanosheets had collapsed into smaller nanoparticles (Figure 5d), and the SAED pattern (Figure 5h) was dominated entirely by blurred Mg(OH)_2_ rings, confirming the near‐complete transformation of the support.

Taken together, the ex situ TEM and operando SXRD results demonstrate that the initially crystalline MgO(111) nanosheets progressively transform into polycrystalline Mg(OH)_2_ domains with diminishing MgO content. Notably, Mg(OH)_2_ becomes the predominant phase after ∼50 min, which aligns with the chronoamperometric data showing an activation period in the first 50 min. This structural transition establishes proton‐hopping pathways through hydroxylated surfaces, indicating that Ru‐Mg(OH)_2_, rather than Ru‐MgO, constitutes the actual active phase for HER in this system.

Spin‐polarized density‐functional theory (DFT) calculations (see Supporting Information for computational details) were carried out to understand how the MgO(111) support transforms under electrochemical conditions and how this affects the Ru catalyst. The polar MgO(111) surface is known to possess significantly higher surface energy than non‐polar surfaces such as MgO(110) due to the intrinsic dipole generated by alternating Mg^2+^ and O^2−^ layers, which makes it thermodynamically less stable. This behavior is well documented in the literature [3, 4, 8, 32, 33]. As shown in Figure S10, the Bader charge analysis further illustrates the pronounced charge redistribution at the Ru‐MgO(111) interface. As evidenced in Figure S11, the H_2_ adsorption energy on MgO (111) (−4.0 eV) is much higher than when it is on the Ru atom (−1.03 eV), which implies a spontaneous migration from the Ru to the support upon dissociative adsorptions of H_2_. The presence of Ru is crucial as it is experimentally proven no reaction will occur in the absence of Ru due to the kinetic stability of MgO. Proton adsorption stabilizes the polar MgO(111) surface by compensating the intrinsic electrostatic dipole. The formation of surface hydroxyl groups therefore reduces the electrostatic instability of the polar surface. In an acidic electrolyte, an applied potential further attracts protons, which compensate for the surface polarity and promote bulk hydrolysis. This explains the extensive MgO(111) to Mg(OH)_2_(001) transition observed experimentally (Figures 3 and 4). A control experiment without applied bias confirmed that MgO remains kinetically stable in the absence of this driving force. We therefore propose that protons are drawn into the layered MgO(111) lattice under bias and that repulsion between protonated oxygen layers help trigger the structural change.

**FIGURE 6:**
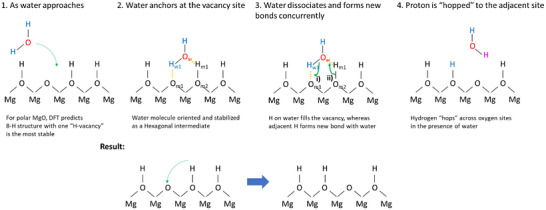
2‐step proton hopping mechanism on 8‐H hydroxylated surface assisted by a water molecule.

Adding Ru makes these tendencies more prominent. Bader charge analysis shows significant charge transfer from Ru to neighboring oxygen atoms on MgO(111), leaving Ru cationic, while Ru on MgO(110) remains nearly neutral. This polarity‐driven charge transfer favors adsorption of cationic species (Ru^2+^ or H^+^) on the O‐terminated MgO(111) surface and explains the strong Ru‐support binding energies calculated for this facet (Figure S12). In contrast, MgO(110) promotes Ru aggregation and shows no comparable transformation. These results match the experimental finding that Ru is highly dispersed and in close contact with MgO(111), conditions that precede formation of the active hydroxide phase. Attempts to prepare Ru‐Mg(OH)_2_ directly was unsuccessful, consistent with the prediction that the hydroxide surface does not stabilize highly dispersed Ru species under ambient conditions. To examine proton conduction, we therefore modeled a hydroxylated MgO(111) surface representing Mg(OH)_2_(001). The most stable configuration obtained from our calculations corresponds to an 8‐H hydroxylated surface containing one surface hydrogen vacancy (Figures S13 and S14). The MgO(111) surface is intrinsically polar due to alternating Mg^2+^ and O^2−^ layers, which generate a strong electrostatic dipole. Progressive proton adsorption compensates this dipole and stabilizes the surface. As shown in Figure S13, increasing hydrogen coverage significantly reduces the plane‐averaged electrostatic potential gradient across the slab, with the 8‐H configuration providing the most effective dipole compensation. In our (3 × 3) surface model, this configuration also leaves one hydrogen vacancy that allows adsorption and migration of additional protons during the reaction. By contrast, a fully hydroxylated 9‐H surface would eliminate all available adsorption sites, preventing further proton hopping. The 8‐H structure therefore represents a physically meaningful model of the protonated MgO(111) surface under acidic HER conditions and is used for the subsequent proton‐transport calculations. A rigorous electrochemical Pourbaix analysis of the Mg–O–H system would provide further thermodynamic insight into surface stability under applied potential and will be explored in future work [34]. When a water molecule approaches this surface, its hydroxyl hydrogen (H_w1_) aligns toward the vacancy while the hydroxyl oxygen (O_w_) forms a hydrogen bond with a neighboring surface hydroxyl (H_m1_). Under an applied potential, a two‐step proton‐transfer sequence occurs (Figure 6):

$$\begin{array}{ccc} \left(i\right)H_{2}O & + & O_{m1}*\to \mathrm{HO}+HO_{m1}*\;\;E_{a}=0.04\;\mathrm{eV}\\ & & \left(\Delta E\;\mathrm{of}\;H_{2}O_{\mathrm{disso}}=0.02\;\mathrm{eV}\right) \end{array}$$

$$\left(\mathrm{ii}\right)\mathrm{HO}+HO_{m2}*\to H_{2}O+O_{m2}*\;\;E_{a}=0.06\;\mathrm{Ev}$$

* O_m1_ and O_m2_ Are Surface Oxygen Sites

Under applied potential, H_w1_ will be dissociated from the water molecule (at an activation energy of 0.04 eV, step (i)) and form a new bond with surface O_m1_. Meanwhile, the neighboring O_m2_─H_m1_ bond will be cleaved (activation energy of 0.06 eV, step (ii)) and the H_m1_ will form new bonding with the hydroxyl oxygen of the water molecule. In other words, the proton can hop across to the adjacent H‐vacancy site in the presence of water. The total energy barrier of only 0.10 eV is far lower than the 0.47 eV required for direct proton hopping, demonstrating that water‐assisted Grotthuss transfer is the preferred pathway and providing a quantitative explanation for the excellent proton conductivity of the electrochemically generated Ru‐Mg(OH)_2_(001) phase.

## 3 Conclusion

In conclusion, we have demonstrated an electrochemically activated magnesium hydroxide catalyst with preferentially exposed (001) facets that deliver outstanding hydrogen‐evolution activity. The material is generated in situ through topotactic hydrolysis of polar MgO(111), as confirmed by in situ synchrotron XRD and XPS, which reveal an ordered transformation from cubic oxide to layered hydroxide. DFT calculations further reveal that proton migration on the hydroxylated Mg(OH)_2_‐like surface proceeds via a water‐assisted two‐step Grotthuss mechanism with a low overall energy barrier of only 0.10 eV, providing a quantitative explanation for the high proton conductivity of the electrochemically generated hydroxide phase and its contribution to the enhanced HER kinetics. After activation, the Ru‐Mg(OH)_2_ catalyst exhibits an approximately 149.5% increase in current density relative to its MgO precursor. This enhancement is associated with the formation of proton‐conductive hydroxylated pathways generated during the electrochemical MgO‐to‐Mg(OH)_2_ transformation. At the same time, the dispersed Ru species provide active sites for hydrogen reduction and facilitate the formation of the hydroxide phase, thereby creating a synergistic environment for efficient proton transport and HER. This work introduces a general strategy for electrochemically driven, facet‐directed conversion of polar oxides into ordered hydroxides, offering a new platform for designing low‐Pt or Pt‐free electrocatalysts and other electrochemical systems where fast proton mobility is essential.

## 4 Experimental Section

### 4.1 Statistical Analysis

All electrochemical measurements were performed using independently prepared electrodes under identical conditions. Data are presented as representative results unless otherwise stated. Where applicable, measurements were repeated at least three times to ensure reproducibility. Data analysis and plotting were carried out using OriginPro (OriginLab Corporation) and Microsoft Excel.

## Author Contributions

J.M. synthesized the materials and performed catalytic experiments. J.M., L.Z., C.T., S.J.D., and T.W.B.L. performed and analyzed the SXRD experiments. A.W.R. acquired TEM images. L.C. and X.‐P.W. performed computational studies. M.M.‐J.L. and S.P.L. performed and analyzed XPS measurements. J.M., S.W., and Y.L. wrote and revised the manuscript. All the authors discussed and contributed further edits to the paper. S.C.E.T. supervised the overall project.

## Conflicts of Interest

The authors declare no conflicts of interest.

## Supporting information




**Supporting File**: advs75242‐sup‐0001‐SuppMat.docx.

## Data Availability

The data that support the findings of this study are available from the corresponding author upon reasonable request.
