# Supplementary material for: Electrochemically Induced Oxide‐to‐Hydroxide Transformation Enables Fast Proton Transport for Enhanced Hydrogen Evolution
Source: Adv Sci (Weinh). 2026 Apr 9:e75242. Online ahead of print. doi: 10.1002/advs.75242 (PMC13334644; doi:10.1002/advs.75242)
Supplement: Supplementary file 1 — Supporting File: advs75242‐sup‐0001‐SuppMat.docx. [file ADVS-9999-e75242-s001.docx]

**Supporting information**

**Electrochemically Induced Oxide-to-Hydroxide Transformation Enables Fast Proton Transport for Enhanced Hydrogen Evolution**

J. Mo et al.

**This file includes:**

Materials and Methods

Figures S1 – S14

Tables S1 – S4

Note S1

References

## **Materials**

The chemicals, reagents, and gases used are as followed: Triruthenium dodecacarbonyl (≥ 99%, Sigma-Aldrich); Magnesium chloride (anhydrous, ≥ 98% Sigma-Aldrich); Magnesium nitrate hexahydrate (ACS reagent, 99%, Sigma-Aldrich); Vulcan carbon (Vulcan XC 72R, FuelCellStore); Benzoic acid (ACS reagent, ≥ 99.5%, Sigma-Aldrich); Sodium hydroxide (ACS reagent, ≥ 97%, Sigma-Aldrich); Tetrahydrofuran (anhydrous, ≥ 99.9%, inhibitor-free, Sigma-Aldrich); Magnesium oxide (ACS reagent, 97%, Sigma-Aldrich); Ethanol (absolute ≥ 99.8% (GC), Sigma-Aldrich); Acetone (99.9%, Sigma-Aldrich); platinum on graphitized carbon (20wt.% loading, Sigma Aldrich), 2-Propanol ($\geq$99.5%, Sigma Aldrich); nafion perfluorinated resin solution (5wt.% in mixture of lower aliphatic alcohols and water, Sigma Aldrich); Sigracet carbon paper (29BC, FuelCellStore); Sulfuric acid (ACS reagent, 95.0-98.0%, Sigma-Aldrich ). H_2_ (99.99%, BOC); Argon (99.99%, BOC); 5% H_2_ in Ar (5% H_2_, 95% Ar, BOC).

## **Material Synthesis**

Ruthenium was loaded on the MgO supports with three different preferentially exposed facets (111), (110) and (100), respectively, then used as a cathode catalyst for the hydrogen evolution reaction. The synthesis involved the preparation of the MgO (111), MgO (110), MgO (100) supports and their corresponding Ru loading catalysts of 20 wt% of Ru-MgO (111), 20 wt% of Ru-MgO (110) and 20 wt% of Ru-MgO (100). And the commercially available 20 wt% Pt/C was supplied by Sigma Aldrich. The catalysts are referred to by their nominal synthesis loading throughout the manuscript and Supporting Information, while the actual metal contents were quantified by ICP-MS and are summarized in Table S1.

#### **Polar MgO (111)**

The MgO (111) support was synthesized via the hydrothermal process modified and optimized from the methods of Wu et al.^[1]^ MgCl_2_ (0.94 g) was dissolved in DI water (30 mL) at room temperature. Benzoic acid (0.12 g), which is acting as a surfactant, was dissolved in another 30 mL of DI water. The above two solutions were then mixed together and stirred for 10 min. NaOH (2M, 20 mL) was added slowly into the mixture drop by drop, forming a white slurry. After aging 1 h at room temperature, the white slurry was transferred to a 100 mL autoclave. The autoclave was then put into the pre-set oven at 180 ^o^C. After 24 h, switch off the oven, then cool down to room temperature. The Mg(OH)_2_ precursor can be collected by centrifuging the slurry obtained by hydrothermal treatment, then washing with DI water for 3 times, followed by drying at 80 ^o^C in a vacuum oven overnight. Finally, the MgO (111) nanosheets were obtained after calcination at 500 ^o^C under air for 6 h.

#### **Non-Polar MgO (110) support**

The MgO (110) support was synthesized by vacuum calcination, following the method of Chen et al.^[2]^ Commercial MgO (500 mg) was boiled in deionized water at 110 ^o^C for 5 h with stirring. The powder was then collected by centrifugation, followed by drying in a vacuum oven at 120 °C for 12 h. After drying, the powder was calcined under vacuum at 500 ^o^C for 6 h.

#### **Non-Polar MgO (100) support**

The MgO (100) support was synthesized via the traditional thermal decomposition method.^[2]^ Mg(NO_3_)_2_  (500 mg) was calcined under compressed air at 500 ^o^C for 5 h.

### ***Ru-loaded MgO catalyst***

In the typical synthesis of the nominal 20 wt% Ru-MgO catalysts, triruthenium dodecacarbonyl (46.8 mg) was dispersed in 30 mL tetrahydrofuran (THF) under sonication for 10 min at room temperature. The above mixture was then transferred to the as-prepared MgO support (200 mg) and allowed to sonicate at ambient temperature for 4 h followed by rotary evaporation to remove the solvent. The obtained powder was then dried at 95 ^o^C in a vacuum oven overnight. After that, the powder was transferred to a temperature-programmed furnace and heated to 300 ^o^C for 4 h under Ar to remove the CO ligand from the Ru precursor.

## **X-ray diffraction (XRD)**

Powder X-ray diffraction patterns were performed using a Bruker D8 Advance diffractometer with LynxEye detector and Cu K_α_ radiation (λ = 1.5406 Å) operating at 40 kV and 25 mA. XRD patterns were collected by scanning from 5° to 90° with a step size of 0.008° and a step time of 25.4 s.

**Transmission electron microscopy (TEM)**

Transmission electron microscopy (TEM) and high-resolution TEM were performed in bright-field mode to investigate the morphology and lattice spacing of the MgO nanostructure.

### **Electrochemical measurement**

The electrochemical measurements were performed with a standard three-electrode system at room temperature using a programmable electrochemical workstation (AUTOLAB PGSTAT320N). The Ag/AgCl (saturated KCl) was used as a reference electrode, while the graphite rod acted as a counter electrode. The catalyst was prepared as a catalyst ink with a concentration of 5 mg/mL in ethanol. Ethanol was selected because it provided reproducible dispersion of the catalyst powder and enabled stable and rapid drying during spray-casting onto carbon paper. The use of a non-aqueous solvent also helps preserve the initial oxide surface prior to electrochemical testing. Unless otherwise stated, all catalysts were prepared using the same solvent composition, Nafion content, and deposition procedure to ensure a consistent comparison.

Briefly, 10 mg of the as-prepared catalyst and 80 uL of 5 wt% Nafion solution were added to 2 mL of ethanol and sonicated for 30 min. Then, the as-prepared catalyst ink was spray-casted onto the carbon paper and dried in air for 30 minutes, yielding a catalyst loading of around 0.5 ± 0.1 mg cm^-2^. The catalyst-casted carbon paper was used as the working electrode. The electrolyte (0.5 M H_2_SO_4_) was purged with Ar for 30 min to get rid of dissolved O_2_ in the solution. Before catalysts were tested for their activity, they were cycled between -0.5 and 1 V vs RHE at 50 mVs^-1^ for at least 10 cycles to clean the surface impurities. Linear scan voltammetry was carried out to determine the HER activity scanning from 0 V to -2.3 V vs RHE at a scan rate of 5 mV s^-1^.

#### **Operando electrochemical-synchrotron XRD analysis**

High-resolution synchrotron X-ray powder diffraction (SXRD) was performed at Beamline I11 in Diamond Light Source, UK. The energy of the incident X-ray flux was set at 25 keV. The tuned energy was chosen to optimize the penetration of the beam through the sample to achieve high contrast (signal-to-noise ratio) and high angular resolution. The synchrotron data were collected by a Pixium RF4343 pixel area detector in transmission geometry. The synchrotron X-ray wavelength was calibrated with the NIST SRMs 674b CeO_2_ standard as λ = 0.49547(1) Å. The high-resolution diffractograms were collected over the 2θ range of 0 to 41.54 °. Each diffraction pattern was collected for 5 min. The electrochemical cell was placed between the penetrated X-ray source and the detector, as shown in Figure S4. The specially designed electrochemical cell was made of PTFE, with sapphire windows in the middle to allow X-ray beam to pass through and diffract from the samples on the working electrode, allowing data to be collected via the pixel area detector in operando during the electrochemical reaction.

**DFT computations**

The spin-polarized DFT calculations were performed using the *Vienna ab initio Simulation Package* (*VASP*) ^[3–5]^ software. The generalized gradient approximation (GGA) based Perdew-Burke-Erzerhof (PBE) functional was used to include the exchange and correlation effect of the valence electrons.^[6]^ The kinetic energy cut-off of 450 eV was used for all calculations. The project-augmented wave (PAW)^[7,8]^ method was used to describe the core–valence electron interactions. Each atom was relaxed until the Hellman−Feynman force criterion was less than 0.01 eV/Å by using the conjugate gradient minimization algorithm. The bulk structure was optimized using a 10 × 10 × 10 Gamma-centered grid of *k*-points mesh. The calculated lattice parameter of the bulk MgO is 4.249 Å, which is very close to the corresponding experimental value of 4.21 Å (calculated from XRD using Bragg’s Law).

The pure MgO (111) surface was modeled using a twelve-layer slab repeated in a 5 × 5 surface unit cell; Symmetric slab model was considered for optimization by fixing the middle four layers to balance the dipole-moment. The *k*-point mesh was sampled using a 1 × 1 × 1 Gamma-centered grid. To avoid interactions between slabs, all slabs were separated by a vacuum gap greater than 15 Å. After complete convergence testing studies, the geometric and electronic structures of the slabs were analyzed. The charge transfer was investigated by Bader charge analysis. A Gaussian smearing with σ = 0.01 eV was used for density of state analysis.

The H adsorption energy (*E*_ads_) on the clean substrate was calculated by:

*E*_ads_ = *E(*_H/sub_) – *E(*_sub_) – ½*E(*_H2_),

where *E(*_H/sub_), *E(*_sub_), and *E(*_H2_) are the DFT energies of the adsorption complex, clean substrate, and gas-phase H_2_ molecule, respectively.

The H adsorption energy on Ru/MgO was calculated by:

*E_ads_* = {*E_(nHRu/MgO)_* – *E_(Ru/MgO)_* – n/2*E_(H2 )_*}/n

where *E_(nHRu/MgO)_*, *E_(Ru/MgO)_*, and *E(*_H2_) are the DFT energies of the adsorption complex, main catalyst, and gas-phase H_2_ molecule, respectively while n is number of H atoms.

The binding energy of Ru atom on MgO surfaces was calculated by:

*E_b_* = E*_(Ru/MgO)_* – E*_(Ru)_* – E*_(MgO)_* (w.r.t. Ru atom)

where *E_(Ru/MgO)_*, *E_(MgO)_*, and E*_(Ru)_* are the DFT energies of the adsorption complex, clean substrate, and Ru atom in gas-phase, respectively.


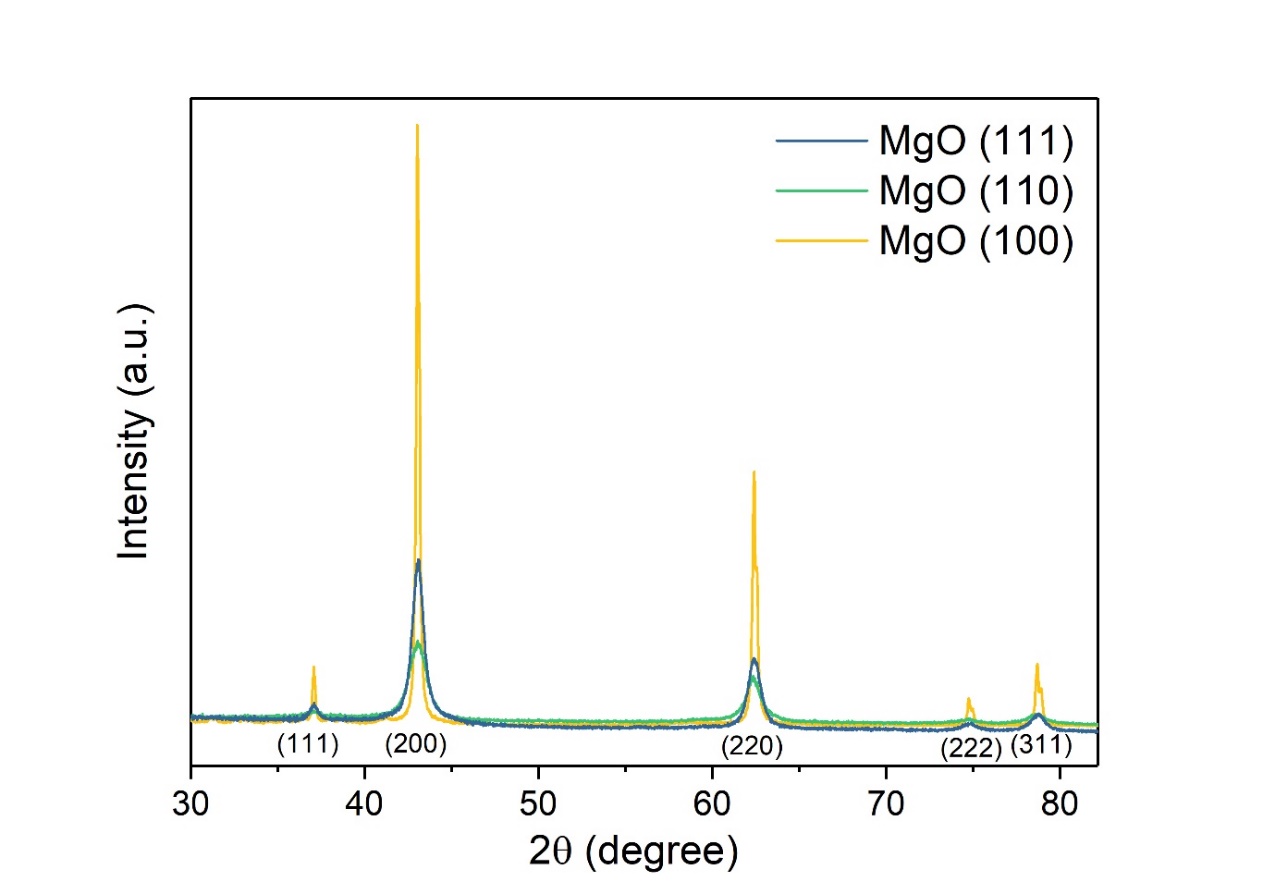


**Figure S1. XRD profile of the MgO (111), MgO (110) and MgO (100) support.**

In Figure S1, it is observed that all the three samples show the highest XRD peak intensity at (200) peak but not the preferentially exposed facet. This can be attributed to the nano-sized nature of the samples in which the orientations are scattered in random directions on the XRD slides with the most thermodynamically stable facets (200) being diffracted. Since XRD reflects the bulk property rather than the surface features, (200) peak is thus the dominant diffracted peak. However, the relative intensity of the exposed facets is in different ratios for each of the three samples. For example, in MgO (100), it displays a relative intensity ratio of 2:1 for facets (200) and (220) whereas a relative intensity ratio of 1:1 in MgO (110) sample. Additionally, the peak width follows the trend of MgO (100) < MgO (111) < MgO (110), which implies a descending order of crystallite size of MgO (100) > MgO (111) > MgO (110) according to the Scherrer equation.


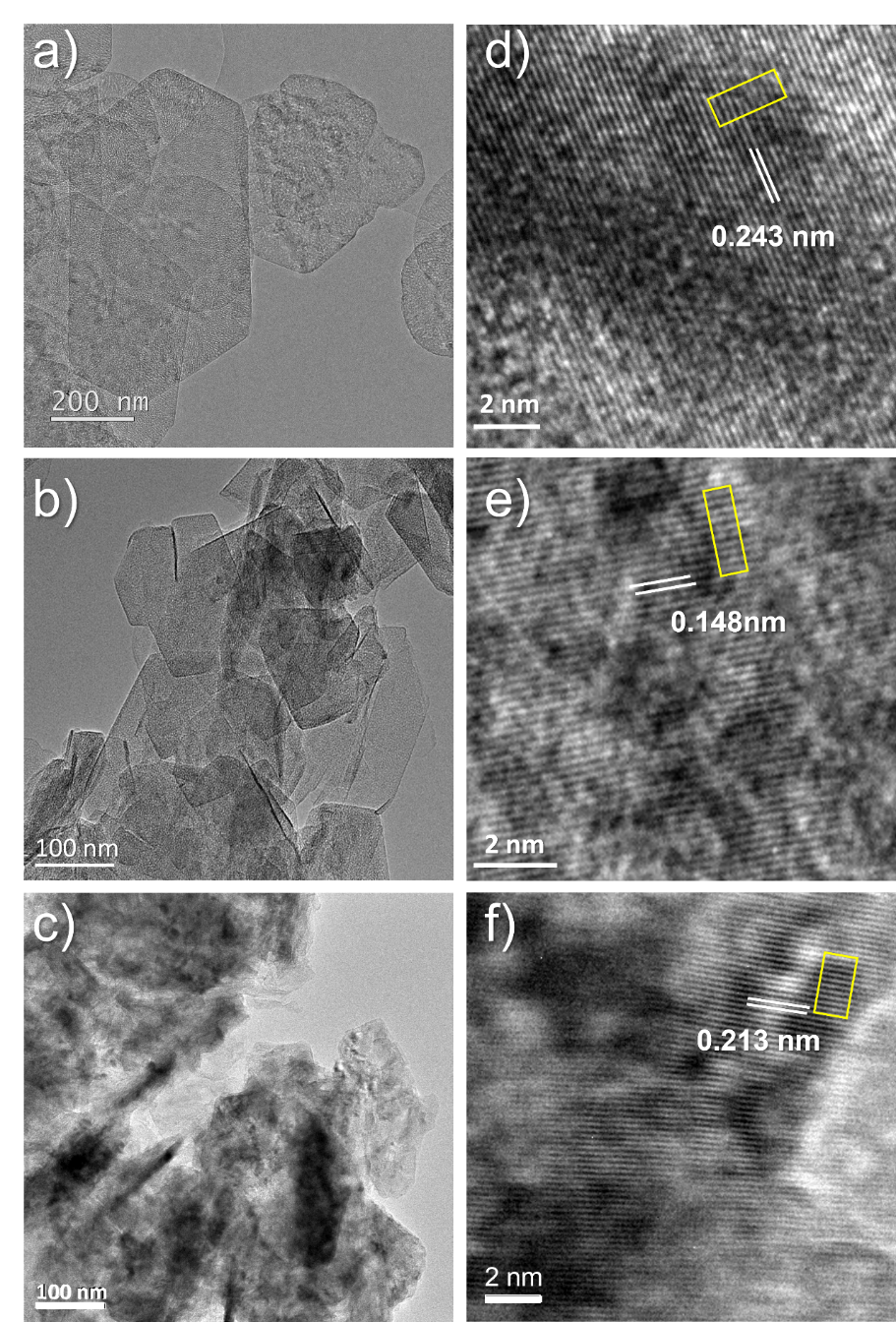


**Figure S2. TEM images** of a) MgO (111), b) MgO (110) and c) MgO (100) supports with their corresponding high resolution TEM d), e) and f) showing the lattice spacings of (111), (220) and (200) facets. The lattice spacing is calculated from the average of 10 spacings where a perpendicular line is drawn across 11 lattice fringes as shown in the yellow square.

The polar MgO (111) sample shows a hexagonal nanosheet structure with a corresponding lattice spacing of 0.243 ± 0.010 nm, which is in good agreement with the literature.^[2,9]^





**Figure S3.** **Chronoamperometric curve** of the nominal 20 wt% Ru-MgO (110) at -1.8 V for 300 min in 0.05 M H_2_SO_4_ electrolyte. Ag/AgCl was used as a reference electrode (RE) and a graphite rod was used as a counter electrode (CE). At the same time, the as-prepared Ru-MgO catalysts were spray-casted onto the carbon acting as a working electrode (WE) and it was placed at the centre of the sapphire window during the operando electrochemical measurement. The concentration of the electrolyte was diluted to 0.05 M in order to minimize the background originating from the solution scattering.


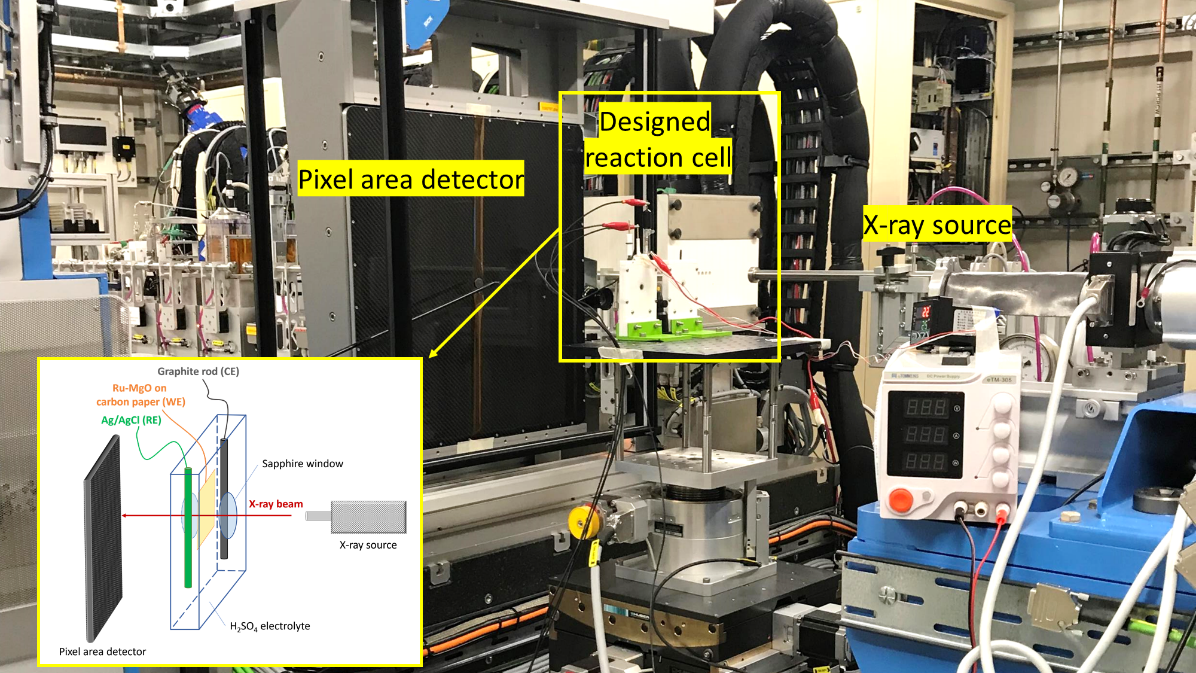


**Figure S4. The operando electrochemical-synchrotron XRD setup in Diamond Light Source, UK, for phase transformation analysis.** The inset is the schematic illustration of the specially designed reaction cell used during the experiment. For operando SXRD measurements, the electrolyte concentration was reduced to 0.05 M H_2_SO_4_ to minimize solution scattering and improve the signal-to-background ratio. This condition was selected to enable structural monitoring under HER conditions, rather than for direct quantitative comparison with polarization data obtained in 0.5 M H_2_SO_4_. Although electrolyte dilution is expected to influence the absolute HER kinetics, the operando SXRD measurements were employed to probe the phase-transition behavior of the catalyst under applied cathodic potential. The observed structural evolution is consistent with ex situ XPS and TEM/SAED results, supporting the conclusion that cathodic operation induces the MgO(111)-to-Mg(OH)_2_ transformation.


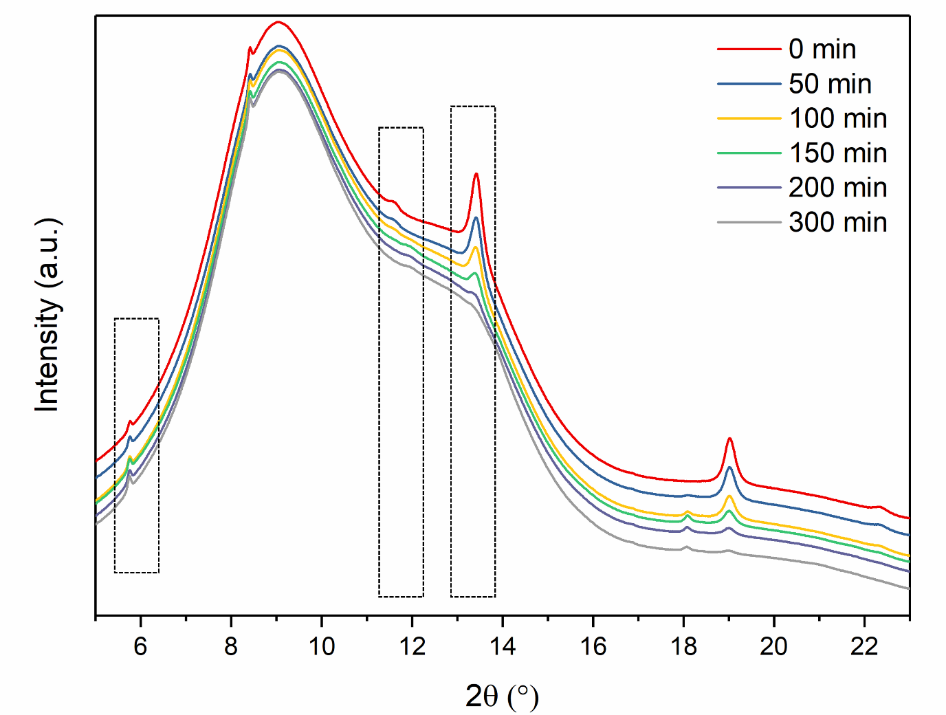


**Figure S5. Operando SXRD patterns of the nominal 20 wt% Ru-MgO (111) sample under -1.8 V with structural changing to Mg(OH)_2._** The diffraction peaks with changing intensity over reaction time in the black dotted boxes (from left to right) were the characteristic peaks of Mg(OH)_2_ (001), MgO (111) and MgO (200), which were the low-angle diffraction peaks for further analysis.


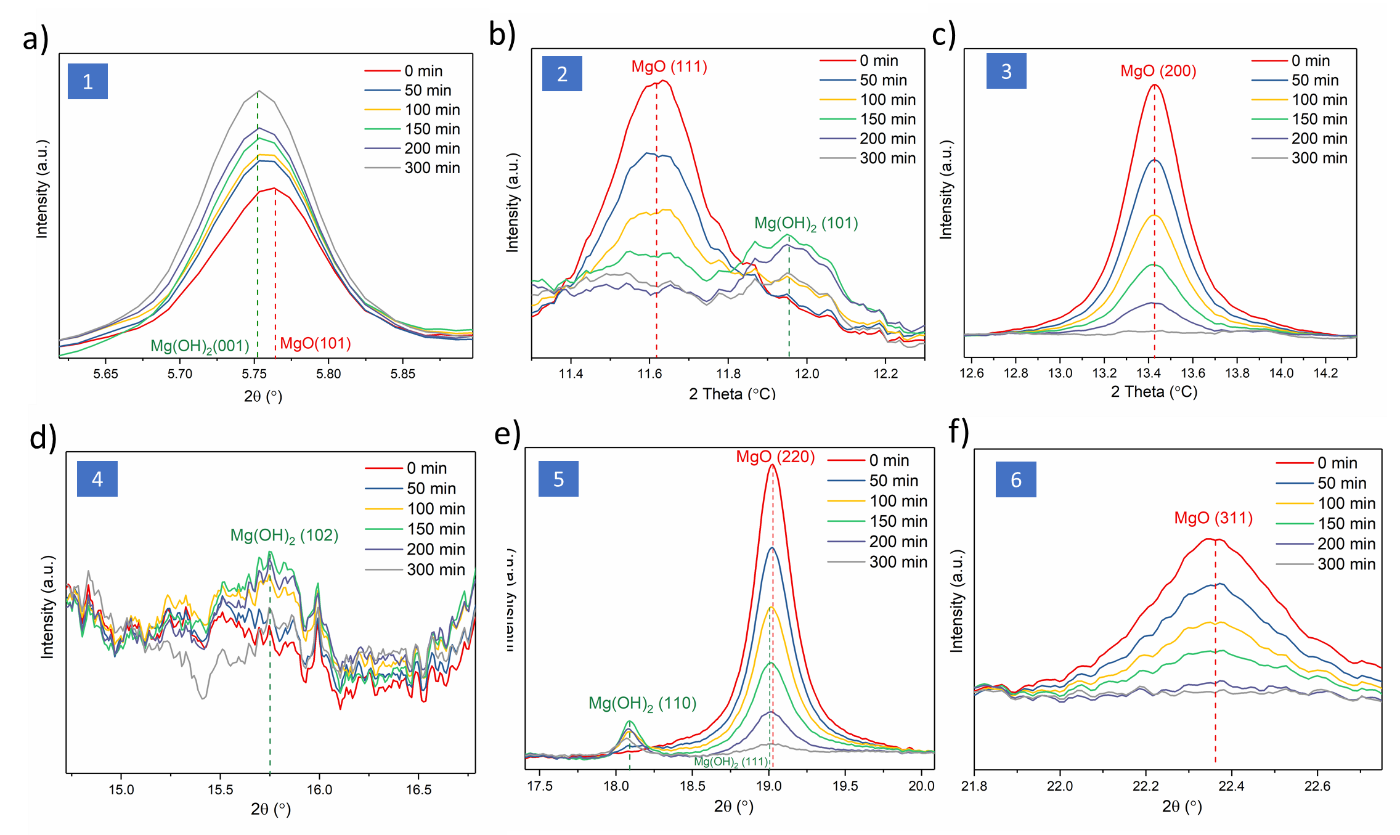


**Figure S6. The enlarged characteristic peaks of MgO and Mg(OH)_2_ in six different 2 theta regions.**





**Figure S7. XRD patterns of the nominal 20 wt% Ru-MgO (111) and after immersion in 0.05 H_2_SO_4_ for 12 h, without electrochemical reaction.**


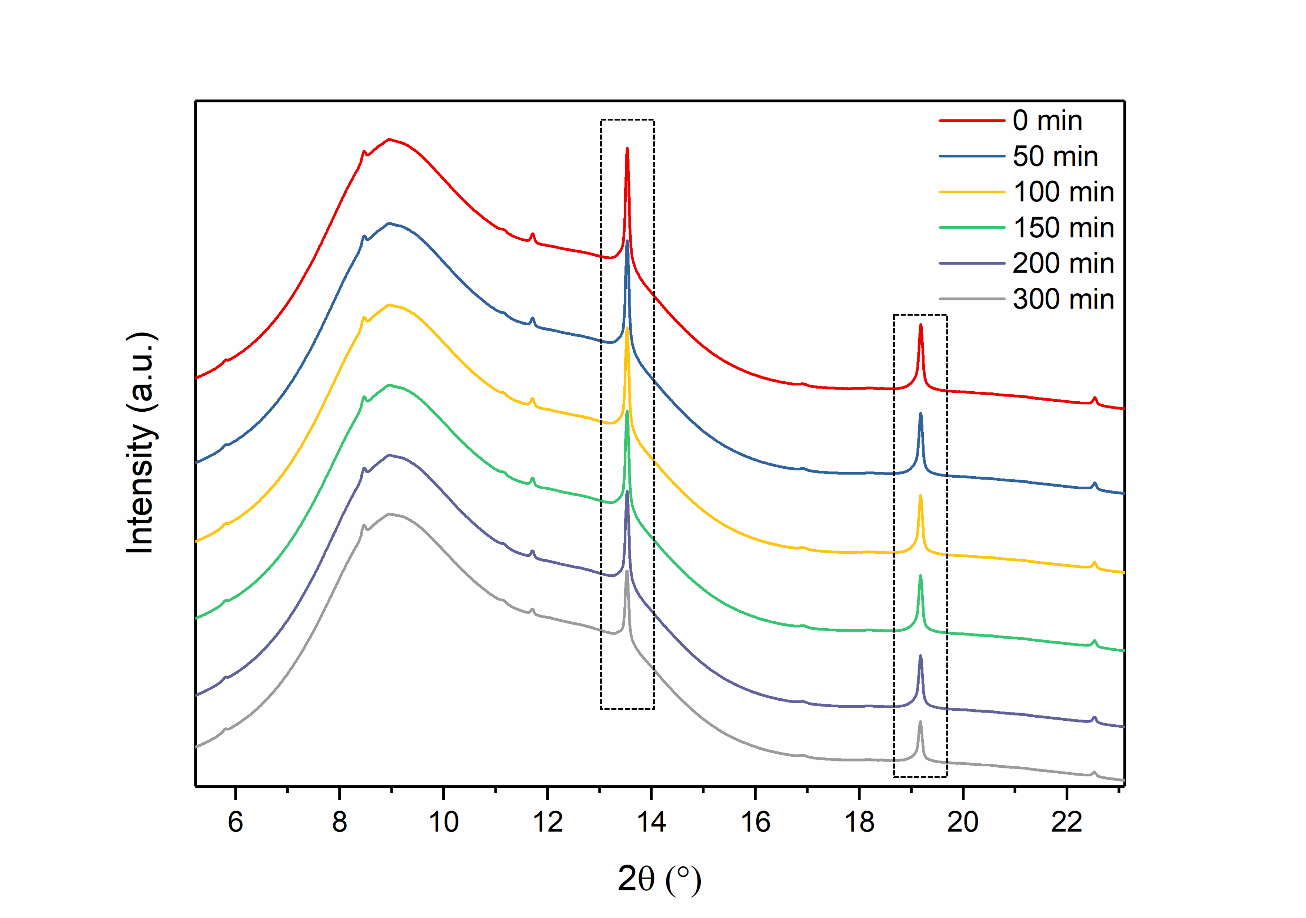


**Figure S8. Operando SXRD patterns of the nominal 20 wt% Ru-MgO (110) sample under -1.8 V_._** The diffraction peak intensities in the black dotted boxes decrease slowly over reaction time, which corresponds to (from left to right) MgO (200) and MgO (220).


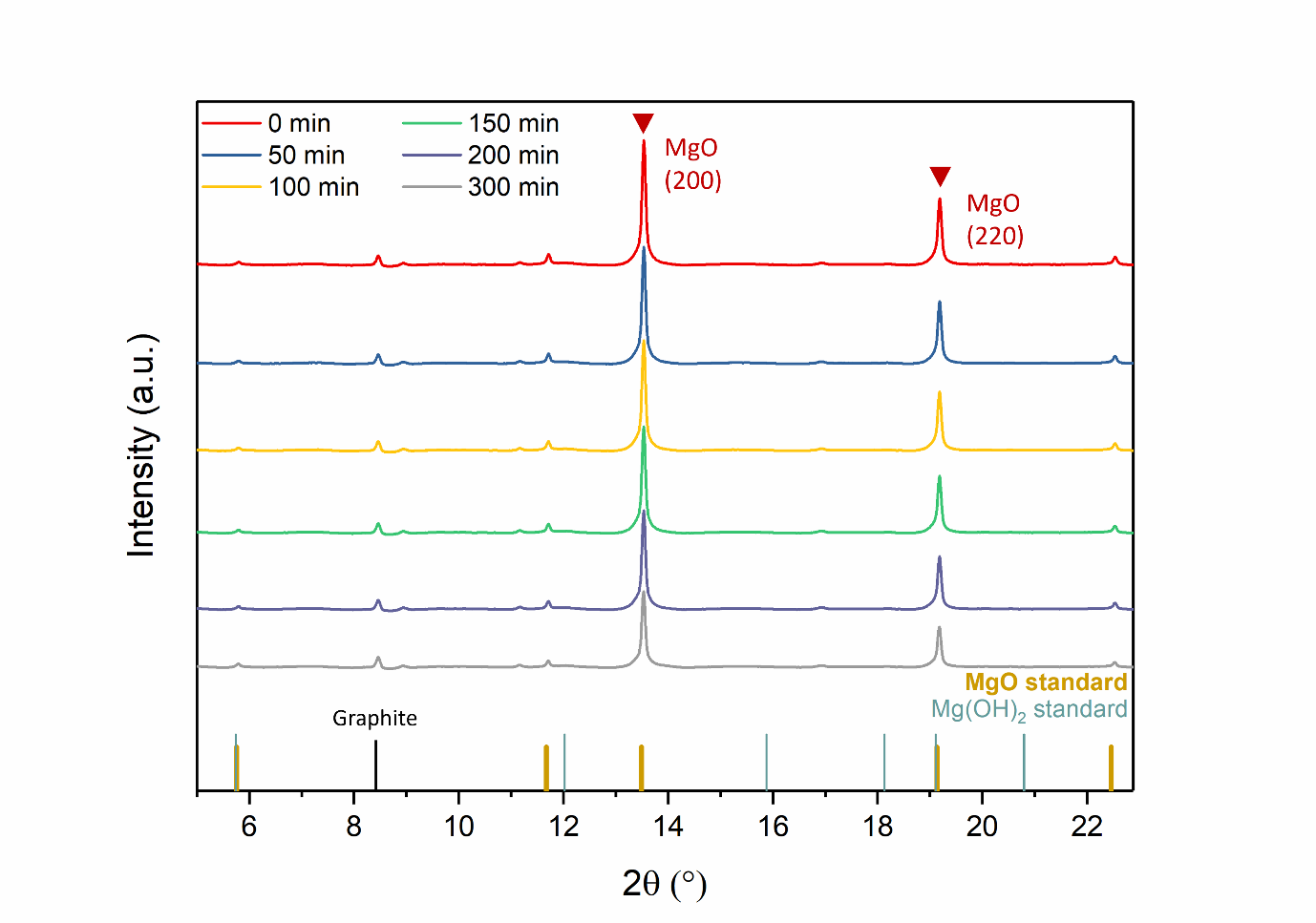


**Figure S9. Background-subtracted operando SXRD patterns of the nominal 20 wt% Ru-MgO (110) sample under -1.8 V.** The reference diffraction patterns of MgO and Mg(OH)_2_ are shown at the bottom for comparison.

**(a)**


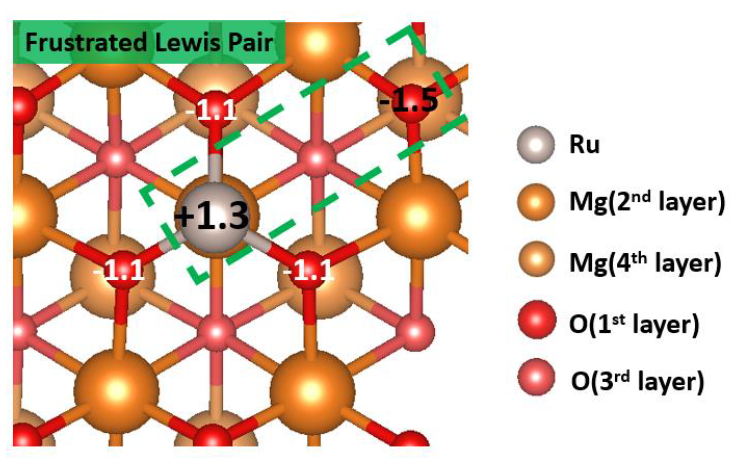


**(b)**


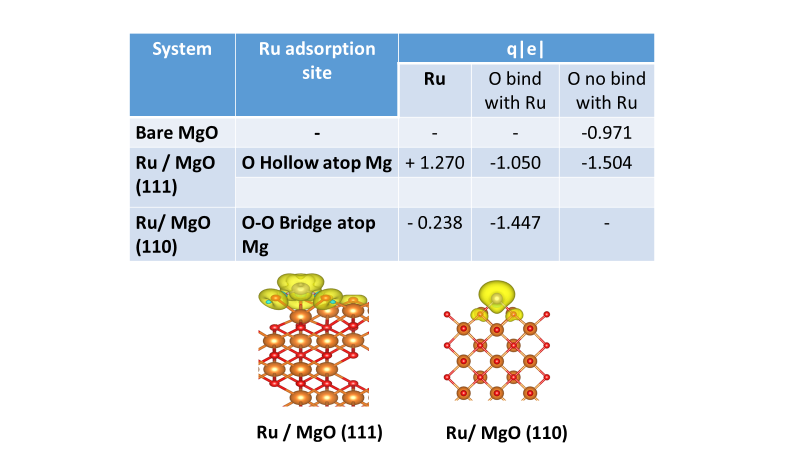


**Figure S10. (a)** The optimized structure of Ru-MgO (111). The number on the atom represents the charge on the surface O and Ru atoms. **(b)** The Bader charge calculation of Ru and O species on MgO(111) and MgO(110).^[10,11]^


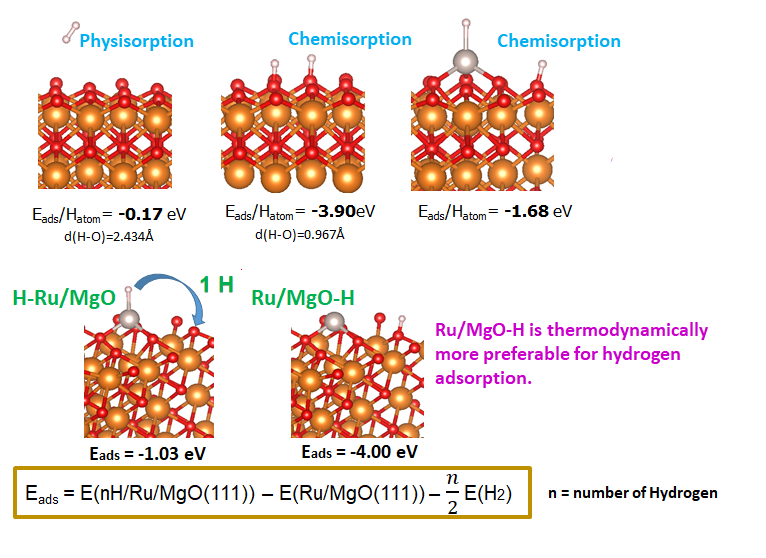


**Figure S11. Ru-MgO (111) gives spontaneous dissociative adsorption for H_2_** (experimentally proven no reaction in the absence of Ru due to the kinetic stability of MgO). ^[10,11]^


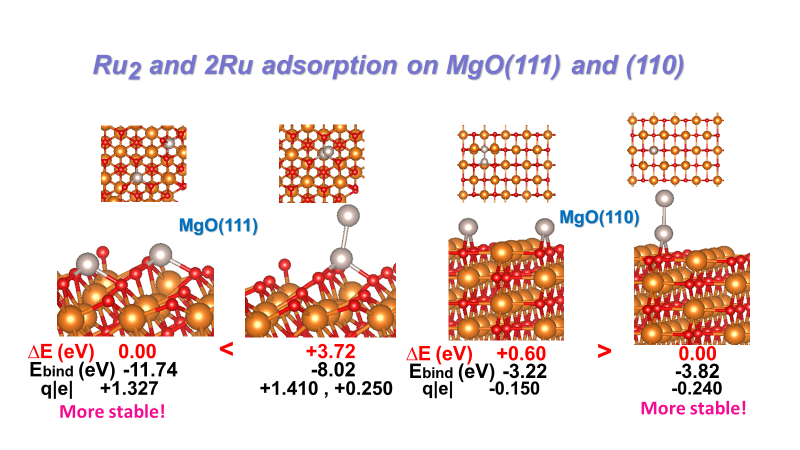


**Figure S12.** Upon placing two Ru atoms on the MgO (111) and MgO (110), it is energetically more favorable (E_bind_) for the two Ru to disperse on MgO (111) due to high surface polarity; whereas the two Ru atoms will prefer aggregation to reduce unfavorable surface contact on MgO (110). ^[10,11]^

*
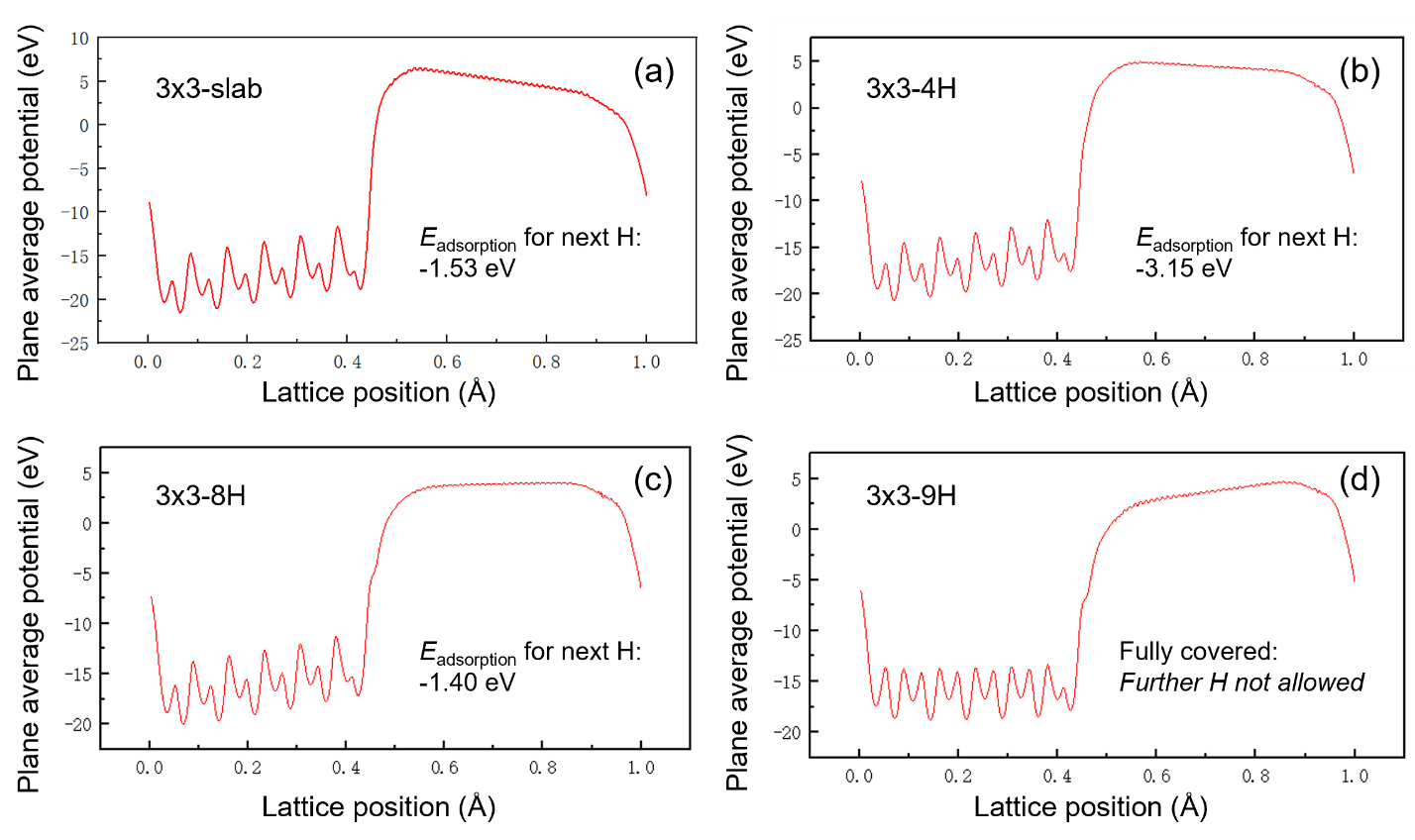
*

**Figure S13.** Plane-averaged electrostatic potential profiles along the surface normal direction for MgO(111) slabs with different hydrogen coverages: (a) 0-H, (b) 4-H, (c) 8-H, and (d) 9-H. The pristine MgO(111) surface exhibits a pronounced electrostatic potential gradient. Increasing hydrogen coverage progressively compensates the surface dipole and reduces the potential gradient. The 8-H configuration provides the most effective dipole compensation while still leaving one surface hydrogen vacancy for additional proton adsorption and migration.


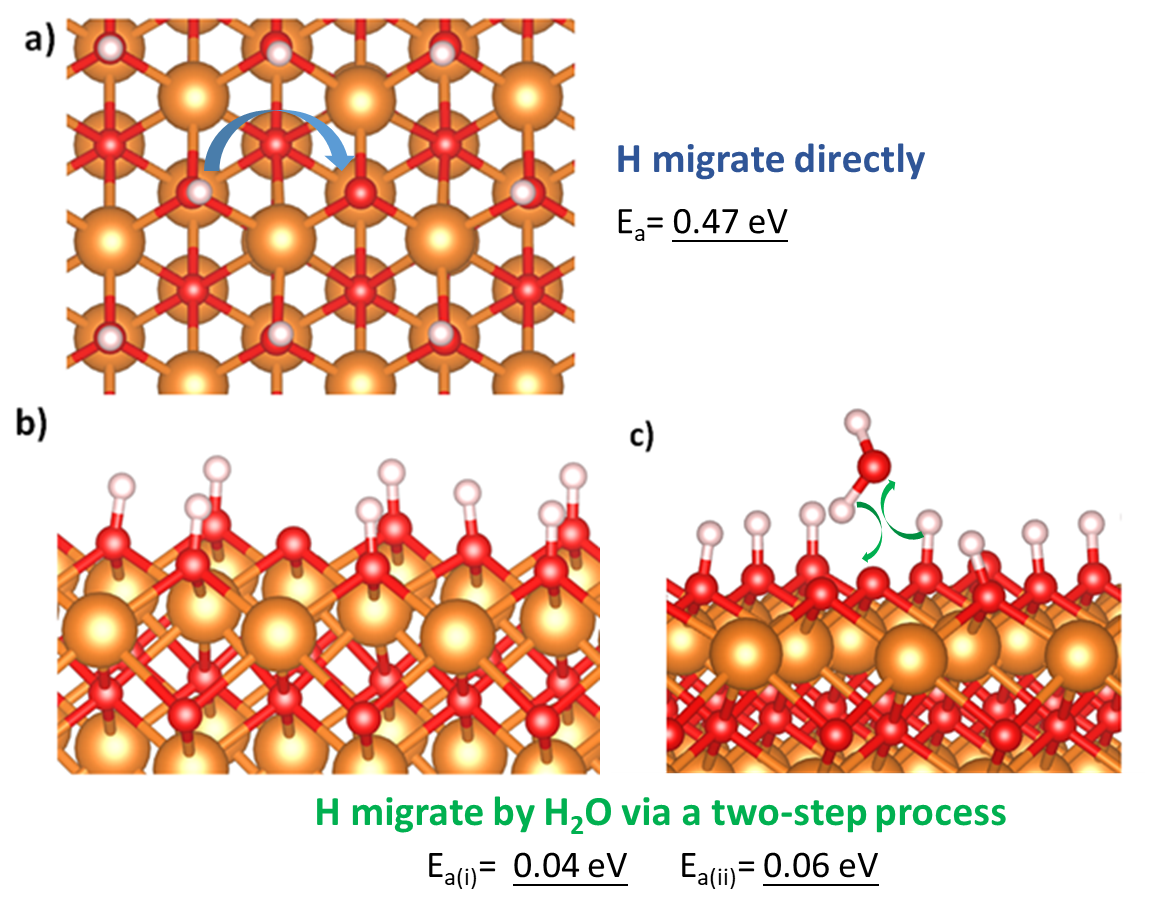


**Figure S14.** (**a**) Direct proton migration on the hydroxylated MgO (111) surface with 8 adsorbed H atoms and 1 H vacancy (same surface feature as Mg(OH)_2_ (001)) in top view. b) Side view of the 8-H MgO (111) surface and **c)** Indirect proton migration on the 8-H MgO (111) surface via H_2_O at side view.

**Table S1** ICP-MS analysis of metals concentrations loaded on different supports.

| Samples | Ru-MgO (110) | Ru-MgO (111) | Commercial Pt/C | Ru/C |
| --- | --- | --- | --- | --- |
| Metal concentrations (wt%) | 15.7 | 14.2 | 20.2 | 21.4 |

The actual metal contents on the different supports were quantified by ICP-MS. The Ru contents are 15.7 wt% for Ru-MgO(110) and 14.2 wt% for Ru–MgO(111), which are lower than the nominal synthesis loading of 20 wt%. The as-prepared Ru/C catalyst contains 21.4 wt% metal, while the commercial Pt/C catalyst used for comparison contains 20.2 wt% Pt.

**Table S2** BET analysis of the three as-prepared MgO supports and commercial activated carbon.

| MgO supports | MgO (111) | MgO (110) | MgO (100) | Activated carbon^[12]^ |
| --- | --- | --- | --- | --- |
| BET surface area  (m^2^ g_cat_^-1^) | 170.9 | 214.1 | 13.4 | 1734 |

As seen in Table S2, the surface area analyzed by BET of MgO (110) is the largest among the three support especially around 20 times higher than that of the MgO (100) supports. The BET surface area trend of MgO (100) < MgO (111) < MgO (110) is in good agreement with the crystallite size as interpreted by the XRD analysis that the MgO (100), with the largest crystallite size, has the smallest BET surface area among the three MgO supports. Additionally, the surface area of the commercial activated carbon is quoted for reference.

**Table S3**. HER mass activity at -2.3 V normalized by the ICP-MS-determined actual metal loading.

| Catalyst | Actual metal content by ICP-MS (wt%) | Total catalyst loading (mg cm^-2^) | Metal loading (mg_metal_ cm^-2^) | *j* at -2.3 V (mA cm^-2^) | Mass activity at -2.3 V (mA mg_metal_^-1^) |
| --- | --- | --- | --- | --- | --- |
| Pt/C | 20.2 | 0.5 | 0.1010 | 495 | 4901 |
| Ru-MgO(111)-A | 14.2 | 0.5 | 0.0710 | 548 | 7718 |
| Ru-MgO(110)-A | 15.7 | 0.5 | 0.0785 | 415 | 5286 |
| Ru/C | 21.4 | 0.5 | 0.1070 | 463 | 4327 |

To account for the deviation between the nominal and actual metal loading, the HER activity at -2.3 V was additionally normalized to the ICP-MS-determined metal content. The metal loading on the electrode was calculated using m_metal_ = m_cat_ x W_metal_, where m_cat_ is the total catalyst loading on the electrode (0.5 mg cm⁻²) and W_metal_ is the actual metal weight fraction measured by ICP-MS. The mass activity was then calculated as j_mass_= j_geo_/m_metal_, where j_geo_is the geometric current density at -2.3 V.

**Table S4** XPS resolved components area ratio.

|  | OH (BE eV) | Intensity | Ratio (%) | O^2-^ (BE eV) | Intensity | Ratio (%) |
| --- | --- | --- | --- | --- | --- | --- |
| 0 min | 532.5 | 27198 | 33.3 | 530.7 | 54598 | 66.7 |
| 10 min | 532.4 | 23984 | 37.1 | 530.7 | 40602 | 62.9 |
| 50min | 532.1 | 34548 | 44.7 | 530.2 | 42691 | 55.3 |
| 100 min | 532.1 | 46291 | 52.9 | 530.1 | 41184 | 47.1 |
| 200 min | 532 | 55831 | 65.1 | 530 | 29911 | 34.9 |

**Note S1 Analysis of faceted MgO materials**

We have previously reported the synthesis and characterization of MgO materials with different exposed facets ^[10,11]^. ^1^H magic-angle spinning (MAS) NMR spectroscopy was employed to investigate the interaction of protons with oxygen anions in the bulk phases of MgO(111), MgO(110), and MgO(100). All MgO samples exhibited a resonance at approximately 0.7 ppm, which is attributed to protons associated with isolated surface hydroxyl groups and physically adsorbed water molecules. In addition, a second resonance at lower field is observed and assigned to bridging hydroxyl protons. Notably, this peak appears at 5.43 ppm for MgO(111), which is significantly downfield-shifted compared with MgO(110) (4.78 ppm) and MgO(100) (4.74 ppm). This pronounced chemical-shift difference indicates stronger proton-oxygen interactions on the polar MgO(111) surface, consistent with preferential proton adsorption on polar facets, analogous to behavior previously reported for ZnO surfaces.

Probe-assisted ^31^P MAS NMR spectroscopy was further conducted to corroborate the surface polarity of MgO(111). Trimethylphosphine oxide (TMPO), a Lewis base, interacts with surface Lewis acidic sites such as Mg^2+^ cations or protonated species, and thus serves as a sensitive probe of surface chemical environments. The ^31^P resonance of TMPO adsorbed on MgO(111) is observed at 45.8 ppm, markedly shifted relative to MgO(110) and MgO(100), both of which exhibit signals at approximately 43 ppm. Considering that physically adsorbed TMPO typically resonates near 41 ppm, the higher chemical shift for MgO(111) reflects stronger Lewis acid-base interactions, providing additional evidence for the enhanced surface polarity of the MgO(111) support.

Detailed experimental results and discussions can be found in our previous papers. ^[10,11]^

**References:**

[1] S. Wu, Y.-K. Peng, T. Chen, J. Mo, A. Large, I. McPherson, H.-L. Chou, I. Wilkinson, F. Venturini, D. (Dave) Grinter, P. Ferrer, G. Held, S. C. E. Tsang, *ACS Catal.* **2020**, DOI 10.1021/acscatal.0c00954.

[2] J. Chen, S. Tian, J. Lu, Y. Xiong, *Appl. Catal. A Gen.* **2015**, *506*, 118.

[3] G. Kresse, J. Furthmüller, *Phys. Rev. B* **1996**, *54*, 11169.

[4] G. Kresse, J. Furthmüller, *Comput. Mater. Sci.* **1996**, *6*, 15.

[5] G. Kresse, J. Hafner, *Phys. Rev. B* **1993**, *47*, 558.

[6] J. P. Perdew, K. Burke, M. Ernzerhof, *Phys. Rev. Lett.* **1996**, *77*, 3865.

[7] P. E. Blöchl, *Phys. Rev. B* **1994**, *50*, 17953.

[8] G. Kresse, D. Joubert, *Phys. Rev. B Condens. Matter Mater. Phys.* **1999**, *59*, 1758.

[9] P. Liu, P. M. Abdala, G. Goubert, M. G. Willinger, C. Copéret, *Angew. Chem. Int. Ed.* **2021**, *60*, 3254.

[10] S. Wu, K. Y. Tseng, R. Kato, T. S. Wu, A. Large, Y. K. Peng, W. Xiang, H. Fang, J. Mo, I. Wilkinson, Y. L. Soo, G. Held, K. Suenaga, T. Li, H. Y. T. Chen, S. C. E. Tsang, *J. Am. Chem. Soc.* **2021**, *143*, 9105.

[11] S. Wu, Y.-K. Peng, A. I. Large, J. Zheng, T. Chen, H. Duan, I. J. McPherson, I. Wilkinson, H.-L. Chou, G. Held, S. C. E. Tsang, *ACS Catal.* **2020**, *10*, 5614.

[12] M. Asadullah, M. A. Rahman, M. A. Motin, M. B. Sultan, *Adsorption Science and Technology* **2006**, *24*, 761.
